# Acoustic Trauma Increases Ribbon Number and Size in Outer Hair Cells of the Mouse Cochlea

**DOI:** 10.1007/s10162-020-00777-w

**Published:** 2020-11-05

**Authors:** Megan B Wood, Nathaniel Nowak, Keira Mull, Adam Goldring, Mohamed Lehar, Paul Albert Fuchs

**Affiliations:** 1 Present Address: Department of Otolaryngology – Head and Neck, Surgery, Johns Hopkins University School of Medicine, 820 Richard Starr Ross Research Building, 720 Rutland Ave, Baltimore, MD 21205 USA; 2 Sutter Instrument, Co. 1 Digital Drive, Novato, CA 94949 USA

**Keywords:** ribbon synapse, outer hair cell, acoustic trauma

## Abstract

Outer hair cells (OHCs) in the mouse cochlea are contacted by up to three type II afferent boutons. On average, only half of these are postsynaptic to presynaptic ribbons. Mice of both sexes were subjected to acoustic trauma that produced a threshold shift of 44.2 ± 9.1 dB 7 days after exposure. Ribbon synapses of OHCs were quantified in post-trauma and littermate controls using immunolabeling of CtBP2. Visualization with virtual reality was used to determine 3-D cytoplasmic localization of CtBP2 puncta to the synaptic pole of OHCs. Acoustic trauma was associated with a statistically significant increase in the number of synaptic ribbons per OHC. Serial section TEM was carried out on similarly treated mice. This also showed a significant increase in the number of ribbons in post-trauma OHCs, as well as a significant increase in ribbon volume compared to ribbons in control OHCs. An increase in OHC ribbon synapses after acoustic trauma is a novel observation that has implications for OHC:type II afferent signaling. A mathematical model showed that the observed increase in OHC ribbons considered alone could produce a significant increase in action potentials among type II afferent neurons during strong acoustic stimulation.

**Supplementary Information:**

The online version contains supplementary material available at 10.1007/s10162-020-00777-w.

## INTRODUCTION

Type II cochlear afferents are uncommon, small-caliber, unmyelinated neurons that project to the brainstem cochlear nucleus (Brown et al. 1988). Their peripheral arbors extend hundreds of microns along the organ of Corti to contact numerous outer hair cells (OHCs) (Spoendlin 1969; Smith 1975; Kiang et al. 1982; Berglund and Ryugo 1987; Brown 1987; Simmons and Liberman 1988). Despite those many contacts, type II afferents respond poorly, if at all, to sound (Robertson 1984; Brown 1994; Robertson et al. 1999; Flores et al. 2015). Consistent with that functional difference, glutamatergic synaptic transmission from OHCs is weak. Individual OHCs release single vesicles with low probability so that summed excitation from the entire pool of presynaptic OHCs is required for action potential initiation (Weisz et al. 2009; Weisz et al. 2012; Weisz et al. 2014). Presynaptic ribbons of OHCs have fewer, disorganized, and variously sized vesicles compared to ribbons of inner hair cells (IHCs) (Weisz et al. 2012). Furthermore, many type II to OHC contacts have no presynaptic ribbons at all (Dunn and Morest 1975; Liberman et al. 1990; Vyas et al. 2017). Immunolocalization of synaptic proteins confirms this arrangement, adding the intriguing observation that, while all type II dendritic contacts label for postsynaptic density proteins, only the half that oppose synaptic ribbons label for GluA2 AMPA receptor subunits (Martinez-Monedero et al. 2016) or KA2 kainate receptor subunits (Fujikawa et al. 2014). This stands in sharp distinction to the potent IHC to type I afferent contacts, virtually all of which include ribbon and postsynaptic GluA2 receptor labeling (Moser et al. 2006; Coate et al. 2019).

Noise-induced hearing loss (NIHL) results from acoustic trauma that damages the cochlear epithelium and spiral ganglion neurons (SGN). Two broad mechanisms are thought to contribute to NIHL following acoustic trauma. First, the damage or death of OHCs causes frank threshold elevation due to lost cochlear amplification. Second, excitotoxic damage to type I afferents confers a more subtle deficit for hearing in noisy settings (Liberman and Kujawa 2017). Damage to type I afferent boutons, accompanied by a loss of presynaptic ribbons from the IHC, is likely due to glutamate excitotoxicity (Kim et al. 2019). Less certain is what leads to the dissolution of presynaptic ribbons. It is well established that IHC ribbons diminish in number with age and after acoustic trauma (Stamataki et al. 2006; Sergeyenko et al. 2013; Kujawa and Liberman 2009). Thus, it may be of interest to examine OHC afferent synapses after acoustic trauma.

In keeping with their essential role in transmission to the brain, and loss after acoustic trauma, ribbon synapses of IHC have been intensively studied. In contrast to the dense afferent innervation of IHCs, OHCs are predominantly postsynaptic to olivocochlear efferents that inhibit electromotility. Only two to three afferent boutons share the OHC’s synaptic space with the much larger efferent synapses. On average, only half those contacts are associated with ribbons (Martinez-Monedero et al. 2016) that drive the occasional release of glutamate from single vesicles (Weisz et al. 2012). Given their low excitability and poor acoustic sensitivity, the alternative suggestion is that type II afferents may serve as cochlear nociceptors, i.e., to detect tissue damage (Flores et al. 2015; Liu et al. 2015). If so, does OHC transmission play a part in this process, given that trauma reduces ribbon number in IHCs? How does acoustic trauma affect the ribbon synapses of OHCs?

In this study, CtBP2 immunolabeling was used to quantify the number of OHC ribbons at several frequencies along the tonotopic axis of the adult mouse cochlea after a threshold-shift-inducing noise exposure (NE). Serial section transmission electron microscopy (TEM) compared the ultrastructure of ribbon synapses in OHCs after acoustic trauma to those of control OHCs. The observed increase in ribbon synapses informed a computational model to estimate the impact on activity of type II afferents during strong acoustic stimulation.

## MATERIALS AND METHODS

**Fig. 1:**
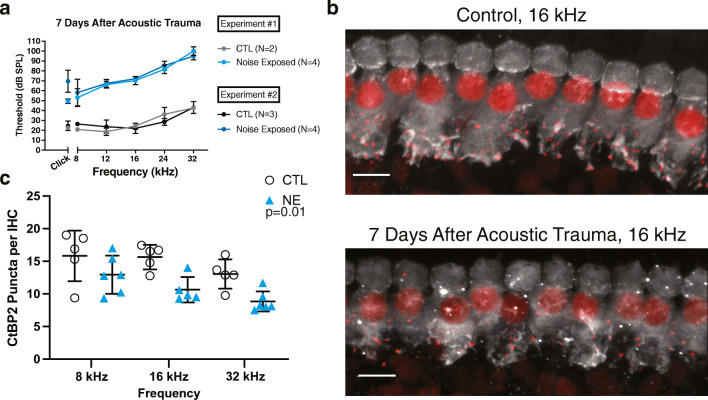
CtBP2 immunopuncta in IHCs decrease in number after acoustic trauma. **a** Average ABR threshold measurements from control and noise-exposed animals 7D after acoustic trauma. Averages of animals from two independent experiments are shown to depict the reproducibility of the noise exposure protocol. **b** Maximum intensity projection (MIP) image of a confocal stack of the 16-kHz region of a whole-mount control (top) and noise-exposed (bottom) organ of Corti. CtBP2—red, myosin VIIa—white; scale bar—10 μm. **c** Quantification of CtBP2 puncta per IHC based on a syGlass reconstruction of a confocal stack of 80 μm of the organ of Corti at each indicated frequency. Each open circle or triangle indicates 1 mouse. When both ears were quantified, the average is represented. *N* = number of mice, *n* = number of ears. 8 kHz CTL: *N* = 5, *n* = 6; 8 kHz NE: *N* = 6, *n* = 6; 16 kHz CTL: *N* = 5, *n* = 6; 16 kHz NE: *N* = 5, *n* = 5; 32 kHz CTL: *N* = 5, *n* = 5; 32 kHz NE: *N* = 6, *n* = 6. Linear mixed model analysis shows a significant effect for noise exposure (*t*_(10.09)_ = − 3.12, *P* = 0.01) and frequency when comparing 8 kHz to 32 kHz (*t*_(21.1)_ = − 3.30, *P* = 0.01). Comparisons between 8 kHz to 16 kHz and 16 kHz to 32 kHz were not significant (8 kHz compared to 16 kHz: *t*_(21.4)_ = 1.03, *P* = 0.57; 16 kHz compared to 32 kHz: *t*_(21.5)_ = − 2.20, *P* = 0.09).

**Fig. 2:**
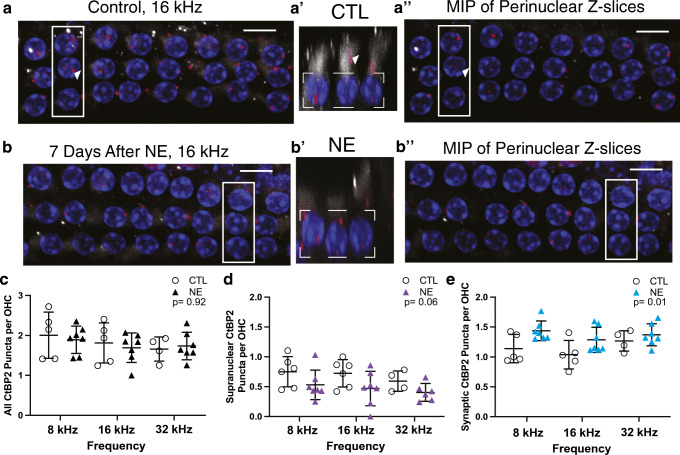
CtBP2 immunopuncta in OHCs increase in number after acoustic trauma. (a) Maximum intensity projection (MIP) image of the entire *Z*-axis of the OHCs from the 16-kHz region of a control animal (CTL). (a′) MIP image of the *ZX* axis from the rectangle indicated in (a). The dashed-line box indicates the *z* region used to generate the MIP image in (a″). (a″) MIP image of the perinuclear *z*-slices from the same confocal stack as (a). The arrowhead points to the same, supranuclear ribbon punctum in (a), (a′), and (a″). (b) MIP image of the OHCs from the 16-kHz region of a noise-exposed animal (NE). (b′) MIP image of the *ZX* axis from the rectangle indicated in (b). (b″) Maximum intensity projection image of the perinuclear *z*-slices indicated in the dashed box of (b′). Nuclei—blue, CtBP2—red, myosin VIIa—white; scale bar—10 μm. (c) Quantification of all CtBP2 immunopuncta per OHC. Each dot or triangle represents the immunopuncta per OHC from an 80-μm region of the organ of Corti for the frequency region indicated from 1 mouse. Linear mixed model analysis showed no significant effect for either noise exposure or frequency. Noise exposure: *t*_(9.983)_ = 0.104, *P* = 0.919. (d) Quantification of supranuclear CtBP2 immunopuncta per OHC. Each dot or triangle represents the immunopuncta per OHC from an 80-μm region of the organ of Corti for the frequency region indicated from 1 mouse. Where both ears were quantified, the average is represented. Linear mixed model analysis shows that the effect of neither noise exposure nor frequency is significant. Noise exposure: *t*_(10.795)_ = − 2.09, *P* = 0.06. (e) Quantification of CtBP2 immunopuncta at the synaptic pole per OHC. Each dot or triangle represents the immunopuncta per OHC from an 80-μm region of the organ of Corti for the frequency region indicated from 1 mouse. Where both ears were quantified, the average is represented. Linear mixed model analysis shows significance for only the effect of noise exposure: *t*_(10.26)_ = 3.41, *P* = 0.01. The interaction between noise exposure and frequency was not significant. *N* = number of mice, *n* = number of ears. 8 kHz CTL: *N* = 5, *n* = 7; 8 kHz NE: *N* = 7, *n* = 10; 16 kHz CTL: *N* = 5, *n* = 7; 16 kHz NE: *N* = 7, *n* = 9; 32 kHz CTL: *N* = 4. *n* = 4; 32 kHz NE: *N* = 7, *n* = 9.

**Fig. 3:**
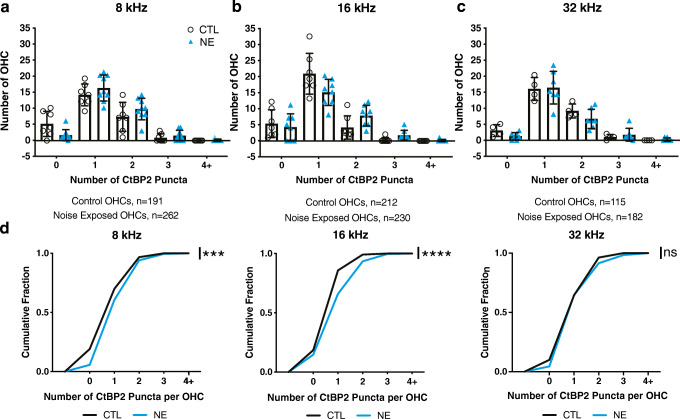
OHCs have an increased probability of more CtBP2 immunopuncta after acoustic trauma. Quantification of number of OHCs with 0 to 4+ CtBP2 immunopuncta at **a** 8 kHz (CTL: *n* = 7, NE: *n* = 9), **b** 16 kHz (CTL: *n* = 7, NE: *n* = 8), and **c** 32 kHz (CTL: *n* = 4, NE: *n* = 7) frequency locations. *n* = number of ears each symbol represents an image from one ear covering 80 μm of organ of Corti from a unique cochlea. **d** Cumulative probability function of CtBP2 puncta per OHC from each frequency region. 8 kHz: ***, Mdn CTL = 1, Mdn NE = 1, *U* = 20,748, *P* = 0.01; 16 kHz: ****, Mdn CTL = 1, Mdn NE = 1, *U* = 19,287, *P* = < 0.001: 32 kHz: ns, Mdn CTL = 1, Mdn NE = 1, *U* = 10,097, *P* = 0.56. *n* = number of OHCs

**Fig. 4:**
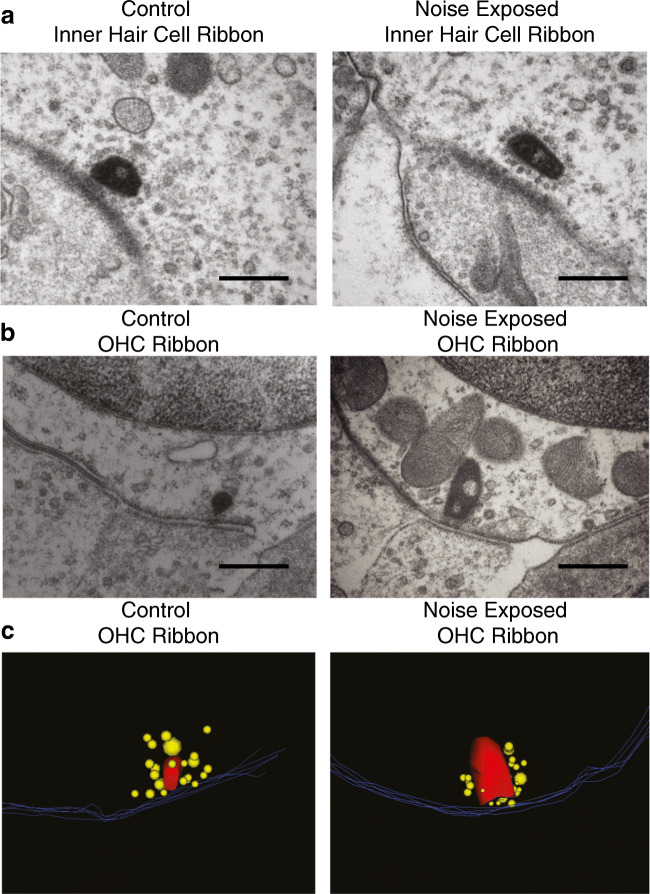
Electron micrographs of OHC ribbon synapses after acoustic trauma show increased volume of ribbon synapses. **a** Example of inner hair cell ribbon synapses from control (left) and after acoustic trauma (right). ×60,000 magnification. Scale bar—400 nm. **b** Example of outer hair cell ribbon synapses from control (left) and after acoustic trauma (right). ×60,000 magnification. Scale bar—400 nm. **c** 3-D reconstructions of ribbons in **b**. Yellow—synaptic vesicles; red—ribbon body, blue lines—OHC cell membrane

**Fig. 5:**
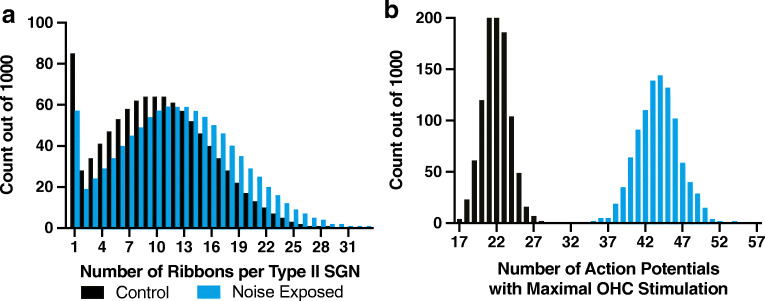
Projected increase in action potential generation in response to maximal OHC stimulation. **a** Histogram of the simulated average number of ribbons presynaptic to the population of type II afferents. Black bars represent estimates derived from control mouse ribbon counts. Cyan bars represent estimates derived from noise-exposed mouse ribbon counts. **b** Histogram of the simulated sum of action potentials generated across the population of type II afferents over 1000 iterations. Black bars represent estimates derived from the simulated number of ribbons per type II afferents in control mice from **a**. Cyan bars represent estimates derived from the simulated number of ribbons per type II afferents in noise-exposed mice from **a**

Mice C56BL/6J (RRID: IMSR_JAX:000664) purchased from Jackson Laboratories were bred and maintained at Johns Hopkins University School of Medicine under the guidance of the Institutional Animal Care and Use Committee (IACUC). Mice were placed on a 12-h light-dark cycle, fed an autoclaved Teklad diet, and housed in cages with automatic water and filtered air until adulthood. All experiments were carried out under protocols approved by the IACUC. Male and female mice were used in the two, independent experiments analyzed in Figs. 1, 2, and 3 and modeled in Fig. 5 to avoid sex-specific variation. The images and data analyzed in Fig. 4 and Tables 1 and 2 are from a separate experiment.

**Table 1:** Quantification of ribbon characteristics from serial-section transmission electron microscopy

	Number of sections	Depth (μm)	Ribbons	Ribbons/micron	OHCs	Ribbons/HC	Ribbon volumes (μm^3^)	Vesicles/ribbon
Control (*N* = 3 mice)	1281	84.69	27	0.32 ± 0.02	39*	0.75 ± 0.59	0.0034 ± 0.0029	22.79 ± 4.82
Acoustic trauma (*N* = 1 mouse)	350	22.75	17	0.75	14*	1.31 ± 0.48	0.0145 ± 0.0194	19.87 ± 11.64
*t* test						*P* = 0.003 *t*_(51)_ = 3.18	*P* = 0.003 *t*_(44)_ = 3.15	*P* = 0.52 *t*_(40)_ = 0.65

Number of ribbons was determined from ×30,000 magnification micrographs. Ribbon volumes and vesicles per ribbon were quantified using ×80,000 magnification micrographs

*N* number of mice

*A minority of OHCs were incomplete and counted as fractions based on the average number of sections per cell. The sum total was rounded off to the nearest whole number

**Table 2:** Quantification of afferent and efferent contacts from serial-section transmission electron microscopy

	OHCs	Total afferents	Total ribbons	Ribbons/cell	Ribbons/micron	Afferents/cell	Ribbons/afferent	Total efferents	Efferents/cell
CTL	11*	11	6	0.55	0.29	1	0.55	21	1.91
NE	14*	26	17	1.21	0.75	1.86	0.65	36	2.57

Quantification was performed on ×30,000 serial micrographs from 295 sections (20.6 μm of tissue) and 350 sections (22.75 μm of tissue) from a control and noise-exposed mouse, respectively

*A minority of OHCs were incomplete and counted as fractions based on the average number of sections per cell. The sum total was rounded off to the nearest whole number

### Noise Exposure(s)

C57BL/6J mice were transferred to a low-noise satellite housing facility from the day before noise exposure through the week after noise exposure until the endpoint for histology. Due to the susceptibility of C57BL/6J mice to age-related hearing loss (Johnson et al. 1997), all experiments were performed with 6-week-old mice. Awake, unrestrained mice were exposed to 110 dB sound pressure level (SPL) white noise for 2 h. Two set-ups were used to perform noise exposure. Animals included in Figs. 1, 2, 3, and 5 were exposed to noise in a reverberant sound-attenuating chamber (58 cm × 40 cm × 30 cm; width, depth, height) with 3 overhead Promaster TW47 1200 W dome tweeter speakers that produced maximum energy in the sound spectrum from 2 to 16 kHz. The speakers were approximately 25 cm above the heads of the mice. Broadband noise was generated by 2 JKTtone and noise generators (KV2 audio, Czech Republic) powered by Neewer nw-100 phantom power sources. The noise generators were connected to 2 Crown Drivecore XLS2502 amplifiers: one driving the two peripheral speakers in input Y mode, the other driving a central speaker in bridge mode. The sound spectra and decibel level were tested in each set-up using a Larson-Davis LXT sound level meter with a ½-in. free-field microphone. Care was taken to measure the sound level at the position of the head of the experimental animals. From the two independent experiments, 5 control and 8 noise-exposed mice were analyzed. Animals prepared for electron microscopy (Fig. 4 and Tables 1 and 2) were exposed to noise on a rotating platform in an acoustic foam-lined sound-attenuating chamber using 2 speakers that had a maximum energy sound spectrum from 4 to 24 kHz for a broadband stimulus. This sound chamber and noise exposure set-up have been previously described (Wu et al. 2020).

### Auditory Brainstem Response

The auditory brainstem response (ABR) system, procedures, and quantification software used for this study have been previously described (Lauer and May 2011; Lina and Lauer 2013). Mice were anesthetized with an intraperitoneal injection of 0.1 cc per 20 g body weight of a mixture of ketamine (100 mg/kg) and xylazine (20 mg/kg) in 14 % ethanol before being placed on a gauze-covered heating pad in the ABR chamber. The animals’ eyes were swabbed with petrolatum-based ophthalmic ointment to prevent corneal ulcers during anesthesia. Subdermal platinum electrodes were placed at the vertex of the head (non-inverting), the left pinna (inverting), and on the left side at the base of the tail (ground). Three hundred repetitions of a click or pure-tone stimulus (10 stimuli/s) were used to generate averaged ABR waveforms. Each tonal stimulus was 5 ms in duration with a 0.5-ms rise and fall time. A Fostex dome tweeter speaker (model FT28D) in a foam-lined chamber was used to present the stimuli to mice 30 cm away. The ABR threshold was defined with custom MatLab software by calculating the averaged peak-to-peak voltage during a 5-ms interval, beginning 1 ms after the onset of the stimulus, compared to the averaged peak-to-peak voltage in a 5-ms window 20 ms after the stimulus. The threshold was determined as the stimulus level where the peak-to-peak response was greater than 2 standard deviations above the electrical noise.

### Fluorescent Immunolabeling and Confocal Microscopy

Cochleas from control and noise-exposed mice were dissected from the temporal bone. Then, the cochleas were fixed with 4 % formaldehyde, which was perfused through the oval window and into the perilymphatic space, exiting the perforated round window. Cochleas were decalcified in 125 mM EDTA for 1 h to overnight, then microdissected into apical, middle, and basal turns. Cochlear microdissections were blocked with a solution of 10 % normal donkey serum, 2 % bovine serum albumin, and 0.6 % Triton X-100 in PBS (2x blocking buffer) for 2 h at room temperature. Primary antibodies were diluted 1:200 in 1x blocking buffer with PBS overnight on a shaker at 4 °C. Mouse IgG1 anti-CtBP2 (Clone 16, BD Biosciences, AB_39943) was used to label ribbon synapses. Mouse IgG2a anti-Tubulin β3 (Clone Tuj1, Biolegend, AB_2313773) was used to label neurons. Rabbit anti-MyoVIIa (polyclonal, Proteus, AB_10015251) was used to label hair cells. Cochlear turns were washed 3x for 10 min in PBS then incubated with secondary antibodies diluted 1:1000 in 1x blocking buffer for 2 h at room temperature. Secondary antibodies used included Alexa-568-conjugated goat anti-mouse IgG1 (Invitrogen, AB_2535766), Alexa-488-conjugated goat anti-mouse IgG2a (Invitrogen, AB_2535771), and Alexa 647-conjugated donkey anti-rabbit IgG (Invitrogen, AB_2536183); 4′6-diamidine-2′-phenylindole dihydrochloride (DAPI, Roche) was used to stain nuclei. Cochlear turns were washed 3x for 10 min in PBS before they were mounted with ProLong Gold Antifade Mountant (Molecular Probes, RRID:SCR_015961). ×10 images of whole turns were imaged using a Zeiss LSM 700 confocal microscope. These images were then analyzed with the Measureline plugin (Eaton-Peabody Laboratories, Mass. Eye and Ear Infirmary) in FIJI to determine the locations of each frequency region. Whole-mount preparations then were imaged using an oil immersion ×40 objective with ×2 digital zoom for a region of 80 μm by 80 μm at each frequency location indicated.

### Quantification of Synapses with Virtual Reality Software

To quantify cochlear ribbon synapses, images were analyzed using ImageJ and syGlass virtual reality software (Istovisio, Inc.) and the Oculus Rift Virtual Reality Headset. syGlass was used to localize the hair cells and cut away the tectorial membrane as necessary. The counting tool in syGlass was used to count two populations of afferent ribbon synapses of whole hair cells: above the nucleus (supranuclear) and below the top of the nucleus (perinuclear, synaptic). Colors were assigned to hair cells and CtBP2 puncta in different locations for reproducibility (IHC nuclei in orange, IHC CtBP2 puncta in yellow, OHC nuclei in green, CtBP2 puncta below OHC nuclei in cyan, CtBP2 puncta above OHC nuclei in violet) (Online Resource 1). Additional analysis on the same images included counting the number of whole OHCs in each tonotopic imaging region with 0, 1, 2, 3, or 4+ CtBP2 immunopuncta. (A separate syGlass experiment was generated so that OHCs with 0 punctum were counted in blue, 1 punctum in red, 2 puncta in orange, 3 puncta in yellow, > 4 puncta in violet). The observer was blinded until all quantification was performed.

### Sample Preparation for Transmission Electron Microscopy

Temporal bones from cochlea of one female, noise-exposed and one male, control mouse were prepared for ultrathin sectioning as previously described (Fuchs et al. 2014). Briefly, temporal bones were perfused through the round window with 1 % osmium (OsO_4_) and 1 % potassium ferricyanide [FeK_3_(CN)_6_] in 0.1 M sym-collidine-HCl buffer. After postfixation for 1 h, tissue was rinsed by perfusion with maleate buffer then decalcified in 5 % EDTA for 48–72 h at 4 °C. Cochleas were embedded in Araldite and sectioned at a 6° angle into 40-μm mid-modiolar sections. Sections were re-embedded in Epon between Aclar sheets. The middle turn was identified, and the organ of Corti was excised from the larger 40-μm section using a rotary microtome. The excised middle turn was embedded onto a blank clock and sectioned at 6° into 65-nm serial sections to create a continuous series for each mouse. The number of serial sections analyzed for each condition (control vs noise-exposed) is indicated in Table 2. The ultrathin sections were then placed on Formvar-coated slot grids for imaging.

### Transmission Electron Microscopy Image Analysis

Electron micrographs were collected at ×30,000–80,000. Identified OHC ribbon synapses were followed and imaged in consecutive serial ultrathin sections. As the images were scanned for OHC ribbons, the row where each OHC was located was noted. All three rows of OHCs are represented in each dataset. Micrographs were imported into Reconstruct software for alignment and analysis (Fiala 2005). Ribbons, vesicles, hair cells, and afferent and efferent plasma membranes were traced and converted into objects. Due to the low number of animals in each group (*N* = 1 control and *N* = 1 noise-exposed animal), two historical control C57BL/6J animals were included in the analysis of Table 1. The preparation of the tissue for these 2 historical controls was the same as described.

### Statistical Model

Mathematical modeling of ribbon and action potential estimates were generated with RStudio software. The numbers of ribbons were generated randomly from a normal distribution centered around the mean number of ribbons per 9 OHCs based on counts from immunofluorescence images analyzed in Figs. 1, 2, and 3. This was replicated 1000 times to create a distribution of estimated ribbons per type II afferent. Given an average probability of release of 0.26 for an individual ribbon, a binomial function was used to determine the probability of at least 7 concurrent excitatory postsynaptic potentials (EPSPs) for each type II afferent neuron, from the estimated number of ribbons (Weisz et al. 2012). Seven concurrent EPSPs are required to reach the action potential threshold. For each set of 1000 neurons, the probability for suprathreshold activation was averaged and multiplied by 1000 to generate the number of action potentials fired across the population.

### Statistics

A linear mixed model analysis was used on datasets where not all animals had both ears analyzed (Figs. 1c and 2c–e). This modeling was based on the chapter “Mixed-Effect Models” from *The R Book* (Crawley 2007) and performed with R studio software. The linear mixed model used noise exposure and frequency as fixed effects with mouse identity as the random effect. The results are presented as (*t*_(degrees of freedom)_ = *x*, *P* = *x*). The *P* value was generated using Satterthwaite’s method. When multiple comparisons were necessary in the case of the three frequency regions, Tukey’s correction for multiple comparisons was performed. *P* values in Fig. 3 were generated from a Mann-Whitney *U* test with Prism 8 software. *P* values in Table 1 were generated using a two-tailed Student’s *t* test with unequal variance. Elsewhere in the text, means and standard deviation are reported as (mean ± standard deviation) as computed with Prism 8.

## RESULTS

To test the effect of acoustic trauma on OHC synapses, 6-week-old C57BL/6J mice were exposed to threshold-shift inducing noise (110 dB for 2 h). The ABR thresholds of mice 7 days after acoustic trauma were reproducibly raised by an average of 44.2 ± 9.1 dB at all frequencies in two experiments (Fig. 1a). A blinded observer quantified ribbon synapses by counting CtBP2 immunopuncta of both OHCs and IHCs. Ribbon synapses were counted in IHCs to determine if this trauma produced the pattern of synaptopathy reported previously (Fernandez et al. 2020). The total number of whole IHCs per image was counted by placing a marker within the nucleus. Figure 1b presents a maximum intensity projection (MIP) confocal image from a control (top) and noise-exposed animal (bottom) in the 16-kHz region of the apical turn to illustrate the loss of CtBP2 puncta in the IHCs 7 days after acoustic trauma. All CtBP2 puncta within the Myosin VIIa staining of the IHC were then counted, and the number of puncta per cell was derived from a ratio of puncta to nuclei (Fig. 1c). The data shown in Fig. 1c are pooled from both noise exposure experiments. The number of CtBP2 immunopuncta in IHCs was lower in the noise-exposed group at all frequencies. A linear mixed model was used to analyze this dataset and showed that the effects of both noise exposure and frequency were statistically significant (NE: (*t*_(10.09)_ = − 3.12, *P* = 0.01; frequency: (8 kHz compared to 32 kHz) *t*_(21.1)_ = − 3.30, *P* = 0.01).

Quantification of CtBP2 immunopuncta from MIP images made from *z*-slices of confocal image stacks can provide misleading data from OHCs. Anti-CtBP2 antibodies label the ribbon synapses at the base of the OHC; however, some labeling exists in the cytoplasm above the nucleus, not associated with the plasma membrane (Online Resource 2). Supranuclear or “misplaced” ribbons have been described previously by immunolabeling of CtBP2 (Liberman and Liberman 2016) and by transmission electron microscopy (TEM) (Sobkowicz et al. 1986). Figure 2 shows MIP images at the 16-kHz region of a control (a, a′, a″) and a noise-exposed (b, b′, b″) mouse. The entire *z*-stack is represented in the re-slice of this confocal stack in a′ and b″. The arrowhead highlights a CtBP2 immunopunctum deep in the cytoplasm above the nucleus, remote from the synaptic zone. This supranuclear ribbon disappears in Fig. 2a″ as this is an MIP image created from a perinuclear *z*-stack represented by the white-dashed box in a′.

To improve accuracy, we used syGlass, a virtual reality visualization program, to quantify the CtBP2 immunopuncta throughout the OHC in our confocal microscopy stacks (see Online Resource 2). The quantification of the ratio of immunopuncta to number of OHCs for three frequency regions was performed by a blinded observer (Fig. 2c–e). The quantification of all the CtBP2 immunopuncta throughout the cell, as represented in Fig. 2a and b, is shown in Fig. 2c. There were no significant differences in the total number of immunopuncta between frequency regions in either the control and noise-exposed groups at 8 kHz CTL: 2.0 ± 0.6, *N* = 5 vs NE: 1.9 ± 0.3, *N* = 7; 16 kHz CTL: 1.8 ± 0.5, *N* = 5 vs NE: 1.7 ± 0.4, *N* = 7; 32 kHz CTL: 1.7 ± 0.3, *N* = 4 vs NE: 1.7 ± 0.3, *N* = 7. The total number of CtBP2 puncta per OHC was not altered by acoustic trauma (*t*_(9.99)_ = 0.10, *P* = 0.92). The number of CtBP2 immunopuncta above and below the nuclei of each OHC was then quantified by viewing the synapses in virtual reality. These counts are presented in Fig. 2d and e, respectively. The number of supranuclear ribbons trends downward after acoustic trauma but just misses statistical significance (Fig. 2d, *t*_(10.79)_ = − 2.09, *P* = 0.06). In contrast, the number of CtBP2 immunopuncta at the synaptic pole of the OHC increased significantly after acoustic trauma. With all OHCs from all regions combined, the average number of synaptic ribbons per OHC (i.e., not supranuclear) was 1.1 ± 0.2 in control cochleas and 1.4 ± 0.2 in cochleas 7 days after acoustic trauma (Fig. 2e, *t*_(10.26)_ = 3.41, *P* = 0.01). The effect of frequency was not statistically significant; however, each frequency region had a slightly different average number of synaptic pole CtBP2 immunopuncta (8 kHz: CTL: 1.1 ± 0.2, *N* = 5, vs NE: 1.4 ± 0.2, *N* = 7; 16 kHz: CTL: 1.1 ± 0.2, *N* = 5, vs NE: 1.3 ± 0.2, *N* = 7; 32 kHz: CTL: 1.3 ± 0.2, *N* = 4, vs NE: 1.4 ± 0.2, *N* = 7). The total number of OHCs was not significantly different between control (28.3 ± 0.6) and noise-exposed (27.8 ± 1.9) cochlear segments (*t*_(12.96)_ = − 0.50, *P* = 0.62). The number of OHCs within each 80-μm segment of the organ of Corti at each frequency region also was not significantly different (8 kHz compared to 16 kHz: *t*_(23.9)_ = − 0.61, *P* = 0.82; 16 kHz compared to 32 kHz: *t*_(26.4)_ = − 0.65, *P* = 0.79; 8 kHz compared to 32 kHz: *t*_(26.4)_ = − 0.15, *P* = 0.99).

To examine the distribution of ribbons per individual OHC, a frequency histogram was generated from the images of each frequency region of the organ of Corti (Fig. 3). The number of synaptic CtBP2 immunopuncta in each OHC was counted, and then the number of OHCs having 0, 1, 2, 3, or 4+ immunopuncta were quantified per image (Fig. 3a–c). The same images used to generate the data in Figs. 1 and 2 were re-analyzed to generate Fig. 3. The distribution of the number of synapses per OHC at each frequency region is shown by mouse in Fig. 3a–c. These data also are plotted as a cumulative probability function at each frequency location in Fig. 3d. The distribution of number of synaptic-region ribbons per OHC is significantly shifted at both 8 kHz and 16 kHz, but not at 32 kHz (Fig. 3d). The overall number of CtBP2 puncta per OHC trends higher at 32 kHz than the 8 and 16 kHz regions in both the control and noise-exposed conditions. This may indicate that a ceiling effect exists in the number of ribbon synapses per OHC toward the base of the cochlea. This could reflect early-onset pathology at the base of the C57BL/6J cochlea (Park, Park et al. 2010). When all frequency regions are pooled together, the cumulative probability function is significantly shifted to the right (*P* = 0.002, Mdn CTL = 1, Mdn NE = 1, *U* = 157,374, CTL: *n* = 575, NE: *n* = 617). In the pooled data, the number of OHCs with 2 ribbon synapses is significantly higher in the noise-exposed group than in control (*t*_(40)_ = 2.686, *P* = 0.01, *t* test not assuming an equal SD).

The ultrastructure of ribbons in control and noise-exposed OHCs was examined with electron microscopy of serial ultrathin sections of a control and noise-exposed mouse cochlea. These samples were age matched to the animals used for immunolabeling. The noise-exposed cochlea and one control cochlea were fixed 7 days after exposure. Two additional datasets from adult C57BL/6J control animals were pooled with the control from this experiment. The acoustic trauma protocol used on the noise-exposed sample had a similar intensity and noise band (4–24 kHz, 110 dB SPL, 2 h) as for the immunolabeling albeit with a different set-up as detailed above in the methods section. In both control and noise-exposed IHCs, synaptic ribbons had large dense bodies surrounded by a well-organized halo of synaptic vesicles (Fig. 4a). As reported previously (Weisz et al. 2012), synaptic ribbons of OHCs were associated with fewer, disorganized, and variously sized vesicles (Fig. 4b). On average, the volume of the ribbon body was significantly larger in noise-exposed OHCs than in control OHCs (Table 1, NE: 0.015 ± 0.019 μm^3^ vs CTL: 0.003 ± 0.003 μm^3^). Synaptic vesicles were counted within 1 μm of the ribbon (Weisz et al. 2012) and did not differ between control and noise-exposed OHCs (vesicles per ribbon: NE: 19.9 ± 11.6 vs CTL: 22.8 ± 14.8) (Table 1).

Consistent with ribbon immunolabeling, the average number of ribbons was larger in the OHCs of the noise-exposed cochlea than in three control cochleas (NE: 1.3 ± 0.5 vs CTL: 0.8 ± 0.6) (Table 1). This distinction held whether accounted on the basis of hair cell number (which required an estimate for fractional OHCs not complete within the series) or simply as a function of tissue depth (i.e., per micron, Table 1). There was no significant difference in ribbons per OHC among the three control cochleas. Immunolabeling in littermates of the same animals, with the antibodies as in Figs. 1 and 2, produced similar results; these immunolabeled images were not identified at a specific frequency location (data not shown).

The synaptic pole of the paired control and noise-exposed cochleas shown in Fig. 4 was further analyzed for the number of type II afferent boutons and medial olivocochlear efferent boutons at the base of each OHC (Table 2). The number of afferent and efferent boutons at the synaptic pole of noise-exposed OHCs is increased in our limited observations from two cochleas. The increase in number of ribbons per OHC in the noise-exposed tissue does not reach a strict 1:1 relationship with the increase in number of afferent boutons resulting in an overall increase in number of ribbons per afferent (Table 2). These observations could indicate sprouting of existing type II afferent neurons after acoustic trauma as part of the changes to the OHC synaptic pole.

The functional consequence of increased OHC ribbons was evaluated by incorporating these histological data into a model of type II afferent activation based on known patterns of synaptic activity (Weisz et al. 2009; Weisz et al. 2012). This asked whether the observed increase of ribbon synapses in noise-exposed cochleas, considered alone, would impact the summed type II afferent activity produced by loud, broadband sound compared to control. Previously published intracellular recordings from type II afferents showed that there are on average 9 functional presynaptic OHCs per type II afferent (range 1–31) (Weisz et al. 2012). These authors also demonstrated that maximal stimulation of an OHC had a one in four chance of releasing a single vesicle during maximal stimulation. Each vesicle causes a 4-mV depolarization on average, thus requiring 7 or more concurrent vesicles for the type II afferent to reach the 25-mV threshold for action potential initiation (Weisz et al. 2012).

To account in silico for the naturally occurring variance in connectivity, a normally distributed dataset was generated around the means for the number of ribbons per 9 OHCs in both control and noise-exposed mice, based on the immunofluorescence counts, while preventing any values less than 1. Each random value thus represents the number of ribbons presynaptic to each single type II afferent. This process was iterated 1000 times to generate the pool of type II afferents (i.e., 8 % of the 12,350 SGNs in one mouse cochlea) (Ehret 1983; Nayagam et al. 2011). This entire process was then repeated 1000 times to approximate variability among cochleas, and so provide an average number of ribbons presynaptic to each type II afferents across all virtual cochleas. Averaging across all 1,000,000 virtual type II afferents, there were 10.2 ± 5.8 and 12.2 ± 6.4 ribbons presynaptic to each type II afferent for control and noise-exposed mice, respectively (Fig. 5a).

For each virtual type II afferent, a binomial function was run to estimate the chance of receiving 7 or more vesicle release events given their number of presynaptic ribbons. The model assumes that all OHCs in the control and noise-exposed group are fully functional and that all presynaptic ribbons are independent (Wu et al. 2016) and have equivalent release properties. For each of the 1000 sets of 1000 neurons, the probability of firing an action potential was averaged and then multiplied by 1000 to estimate the number of action potentials produced by the entire population for a maximal stimulus such as one due to a loud, broadband sound. The average number of action potentials for the entire population of type II afferents was 21.9 ± 1.8 in the control condition and 43.7 ± 2.9 for the noise-exposed condition (Fig. 5b). The two resulting distributions did not overlap. Every estimate in the noise-exposed group was larger than the largest control group estimate (Fig. 5b). Thus, the global 20 % increase in OHC ribbon count observed in the noise-exposed mice is estimated to cause a 99 % increase in action potentials in response to maximal stimulation of OHCs across the entire population of 1000 virtual type II afferents.

## DISCUSSION

The synaptic arrangements of inner and outer hair cells differ markedly. Type I afferents transmit the details of acoustic signals via a single dendritic terminal with a single IHC. All type I dendrites express postsynaptic density markers and glutamate receptor subunits and are associated with presynaptic ribbons in the hair cell. In contrast, type II afferents transmit limited, if any, acoustic information despite terminal contacts with 16 OHCs on average (rat, apical, cochlea) (Weisz et al. 2012; Jagger and Housley 2003). While all type II dendritic terminals express postsynaptic density proteins, approximately half of the type II afferent bouton endings are not associated with a presynaptic ribbon (Martinez-Monedero et al. 2016). Furthermore, only “ribbon-associated” type II dendrites express the GluA2 receptor subunit (Martinez-Monedero et al. 2016; Vyas et al. 2017). The “ribbonless” OHC to type II afferent contacts are reminiscent of “silent synapses” in the central nervous system that act as a reservoir of plasticity for activity-induced upregulation (Nakayama et al. 2005).

The present study interrogated the relationship between activity-dependent modulation of OHC ribbons and IHC ribbons after acoustic trauma. Noise exposure producing a 40-dB threshold shift resulted in a significant increase in the number of synaptic ribbons in OHCs. In these same cochleas, IHC ribbons decreased in number as described previously (Stamataki et al. 2006; Boero et al. 2018; Fernandez et al. 2020; Kujawa and Liberman 2009). Thus, not only do the synaptic arrangements of inner and outer hair cells differ, but they have an opposite response to acoustic trauma. This has not been reported previously for OHCs or other mammalian hair cells but may have a parallel in zebrafish where synaptically silent lateral line hair cells increase transmitter release after trauma (Zhang et al. 2018).

These observations were aided by the ability to quantify CtBP2 staining in 3-D virtual reality and by supporting ultrastructural studies using TEM. Both methods showed that ribbons at the synaptic pole of the cell increased in number after acoustic trauma. However, this effect was obscured when the total number of CtBP2 puncta per OHC was counted, due to variability in the number of puncta located in the cytoplasm between the nucleus and cuticular plate (Fig. 2c). Supranuclear CtBP2 immunopuncta have unknown functions; however, they appear far from the synaptic zone of the OHC and 3-D visualization clearly locates many deep within the cytoplasm. Nonetheless, since Myosin 7a rather than a specific membrane marker was used, it cannot be asserted that every supranuclear CtBP2 punctum is non-synaptic. At the same time, ultrastructural studies in several species, including mice, invariably show afferent synapses clustered at the synaptic pole of the OHCs, largely below the nucleus (Hashimoto and Takasaka 1989; Liberman et al. 1990; Sato et al. 1999; Fuchs et al. 2014). Immunolabeling of postsynaptic proteins in type II afferents would help to determine the functionality of both supra- and subnuclear ribbons in noise-exposed IHCs if immunolabeling were as effective in mouse tissue as in rats (Martinez-Monedero et al. 2016).

Exposure to damaging noise can be graded to produce different pathological outcomes. Noise-induced OHC pathology that causes temporary to permanent threshold shifts result from OHC damage or apoptosis (Hu et al. 2000; Bohne et al. 2007). An increase in ribbon synapses in OHCs after acoustic trauma could be postulated to be a mechanism of compensation. However, it should be noted that the acoustic trauma used here did not result in significant OHC loss, particularly in the most apical cochlea where the increase in ribbon number was most pronounced. Therefore, increased OHC ribbon synapse number may be a response to maximal acoustic stimulation, rather than an effect of cell damage. Lastly, repeated noise exposure has cumulative effects on hearing (Wang and Ren 2012). It is not yet known how long the change in number of OHC ribbon synapses may persist or whether additional noise exposure would prolong this effect. In this context, it may be of interest that ribbon numbers did not increase in OHCs of the 32-kHz region (Fig. 3) and above (data not shown). This may be a ceiling effect due to trauma from ambient noise exposure in the more vulnerable basal portions of the cochlea even at 6 weeks of age.

In addition to an increase in ribbon number, the traumatized OHCs included some very large ribbons so that the average size was significantly greater than that of control OHCs (Table 1). Intriguingly, enlarged ribbons also were documented in the IHCs of aged, deaf C57BL/6J mice (Stamataki et al. 2006). The functional significance of ribbon enlargement remains to be determined, as do the factors that dictate ribbon size. Studies in zebrafish have begun to uncover potential molecular mechanisms regulating ribbon size. These have shown an interplay between calcium influx, mitochondrial NAD/NADH, and ribeye self-assembly through an NADH binding site (Wong et al. 2019). Future studies should explore the effect of acoustic trauma on calcium buffering and mitochondrial activity in cochlear hair cells.

A potential role for increased type II afferent neuron activity relates to the condition of hyperacusis whereby louder sounds can provoke pain (Pienkowski et al. 2014). Like somatic hyperalgesia, hyperacusis is associated with peripheral damage. Somatic hyperalgesia involves increased peripheral excitability as well as plasticity of central connections (Treede et al. 1992). Therefore, an important question for the present observations remains: is the observed increase in OHC ribbon number meaningful? The model presented in Fig. 5 shows the projected impact of increased ribbon number on the activity of the entire population of type II afferents as might occur during maximal broadband acoustic stimulation. Despite the modest increase in the number of OHC ribbons, the model predicted a doubling in action potentials generated by type II afferents. There are, however, limitations to the statistical model. First, it was assumed that before and after damage there are equal numbers of functional, healthy OHCs. While there was not a significant degree of OHC loss following the noise exposure protocol, damage to stereocilia could reduce mechanotransduction. If true, the model would overestimate the number of functional synaptic inputs. Secondly, the dataset used to generate the parameters for the model was taken from a study in young, prehearing rats (Weisz et al. 2012). The number of ribbons in OHCs declines soon after birth, with that change essentially complete in the first postnatal week. Also, species differences and the effects of maturation could alter the number of presynaptic OHCs, their release properties, or the intrinsic excitability of type II afferents. The limited observation that the number of afferent boutons is increased following acoustic trauma could indicate type II:OHC connectivity is altered after acoustic trauma. Thus, any of these parameters could change following noise trauma and are a potential avenue of future research into the effects of damage on type II afferent function. Nonetheless, the present work provides evidence that OHC ribbon numbers increase after acoustic trauma and that change alone could significantly increase sound-evoked type II afferent signaling to the brain.

This proposed increase of hair cell to afferent signaling is just one of many changes that may result from acoustic trauma. For instance, OHC hair bundles may be damaged by sound; in vitro, tip link regeneration takes 48 h (Indzhykulian et al. 2013). Here, we have examined OHC ribbon synapses 7 days after noise exposure. The model presented assumes that OHCs retain normal sensitivity. Also, it is known that acute tissue damage initiates spreading waves of activity among supporting cells via the release of ATP (Sirko et al. 2019). Type II afferents are excited by ATP (Liu et al. 2015), and could become sensitized in the presence of ATP as is observed in somatic nociceptors (Kress and Guenther 1999; Tominaga et al. 2001). Tissue damage caused by acoustic trauma also induces a prolonged inflammatory response. Specifically, SGNs express cytokines such as IL-6 and a large number of macrophages infiltrate the cochlea (Fujioka et al. 2006; Kaur et al. 2019). Both cytokine expression and immune cell activation are correlated with persistent hyperalgesia in models of inflammatory pain (Dina et al. 2008; Goncalves et al. 2019). While many questions still remain unanswered, the current work provides the surprising observation that the synaptic function of OHCs can be increased after acoustic trauma, providing one component of enhanced cochlear excitation. Further functional studies will help elucidate how this phenomenon of increased OHC ribbon number may affect acoustic sensation.

## Supplementary Information

ESM 1 (MOV 48491 kb)

ESM 2 (MOV 12232 kb)

## Data Availability

Raw data used to form the conclusions of this publication are available upon request.
